# International burden of cancer deaths and years of life lost from cancer attributable to four major risk factors: a population-based study in Brazil, Russia, India, China, South Africa, the United Kingdom, and United States

**DOI:** 10.1016/j.eclinm.2023.102289

**Published:** 2023-11-15

**Authors:** Harriet Rumgay, Citadel J. Cabasag, Judith Offman, Marianna de Camargo Cancela, Anton Barchuk, Prashant Mathur, Shaoming Wang, Wenqiang Wei, Peter Sasieni, Isabelle Soerjomataram

**Affiliations:** a Cancer Surveillance Branch, International Agency for Research on Cancer, Lyon, France; b School of Cancer & Pharmaceutical Sciences, Faculty of Life Sciences & Medicine, King's College London, London, UK; c Centre for Prevention, Detection and Diagnosis, Wolfson Institute of Population Health, Queen Mary University of London, London, UK; d Division of Cancer Surveillance and Data Analysis, Brazilian National Cancer Institute (INCA), Rio de Janeiro, Brazil; e Institute for Interdisciplinary Health Research, European University at St. Petersburg, St. Petersburg, Russia; f ITMO University, St. Petersburg, Russia; g Indian Council of Medical Research - National Centre for Disease Informatics and Research, Bengaluru, India; h National Central Cancer Registry, National Cancer Center/National Clinical Research Center for Cancer/Cancer Hospital, Chinese Academy of Medical Sciences and Peking Union Medical College, Beijing, China

**Keywords:** Cancer mortality, Years of life lost, Risk factors, Cancer prevention, Cancer control

## Abstract

**Background:**

We provide a comprehensive view of the impact of alcohol consumption, tobacco smoking, excess body weight, and human papillomavirus (HPV) infection on cancer mortality and years of life lost (YLLs) in Brazil, Russia, India, China, South Africa, the United Kingdom (UK), and United States (US).

**Methods:**

We collected population attributable fractions of the four risk factors from global population-based studies and applied these to estimates of cancer deaths in 2020 to obtain potentially preventable cancer deaths and their 95% confidence intervals (CIs). Using life tables, we calculated the number and age-standardised rates of YLLs (ASYR).

**Findings:**

In Brazil, Russia, India, China, South Africa, the UK, and the US in 2020, an estimated 5.9 million (3.3 million–8.6 million) YLLs from cancer were attributable to alcohol consumption, 20.8 million (17.0 million–24.6 million) YLLs to tobacco smoking, 3.1 million (2.4 million–3.8 million) YLLs to excess body weight, and 4.0 million (3.9 million–4.2 million) YLLs to HPV infection. The ASYR from cancer due to alcohol consumption was highest in China (351.4 YLLs per 100,000 population [95% CI 194.5–519.2]) and lowest in the US (113.5 [69.6–157.1]) and India (115.4 [49.7–172.7). For tobacco smoking, China (1159.9 [950.6–1361.8]) had the highest ASYR followed by Russia (996.8 [831.0–1154.5). For excess body weight, Russia and the US had the highest ASYRs (385.1 [280.6–481.2] and 369.4 [299.6–433.6], respectively). The highest ASYR due to HPV infection was in South Africa (457.1 [453.3–462.6]). ASYRs for alcohol consumption and tobacco smoking were higher among men than women, whereas women had higher ASYRs for excess body weight and HPV infection.

**Interpretation:**

Our findings demonstrate the importance of cancer control efforts to reduce the burden of cancer death and YLLs due to modifiable cancer risk factors and promote the use of YLLs to summarise disease burden.

**Funding:**

10.13039/501100000289Cancer Research UK.

**Research in context**

**Evidence before this study**

Previous studies have estimated the impact of risk factors on cancer incidence and sometimes mortality but few have provided an analysis incorporating years of life lost (YLLs). YLLs can be used to quantify the societal impact of premature deaths from diseases. We aimed to fill this gap in the literature by combining estimates of cancer deaths attributable to risk factors with measures of YLLs to provide a more comprehensive view of the impact that these different risk factors have on societies.

**Added value of this study**

Our study examines the impact of alcohol consumption, excess body weight, human papillomavirus infection, and tobacco smoking on mortality from cancer and YLLs in Brazil, Russia, India, China, South Africa, the United Kingdom, and the United States. Disparities in the burden of YLLs between countries and between men and women largely reflect differences in risk factor exposure and cancer mortality in each population. Our study emphasises the strength of using a common methodology and data sources to compare the impact of risk factors on cancer mortality between countries. Through this study we demonstrate that with publicly available data, public health advocates can construct a valuable tool to aid public health decision-making. We examined seven countries and four risk factors only, but this exercise could be undertaken for countries across the world where population data are available and should be extended to further risk factors.

**Implications of all the available evidence**

Primary prevention of cancer is key in reducing cancer mortality and its impact on society internationally. Together with the existing evidence, our observations should be used to demonstrate the importance of implementing further cancer control efforts to reduce the preventable burden of cancer mortality due to modifiable cancer risk factors.

## Introduction

Cancer poses a major burden of disease and death globally. Yet many causes of cancer are avoidable through prevention of exposure to modifiable risk factors such as tobacco use, alcohol consumption, excess body weight, and infections from some pathogens.^1^, ^2^, ^3^ There are various methods to measure the magnitude of the impact of these risk factors on cancer burden. For example, the number of potentially preventable cancer cases and deaths can be estimated using population attributable fractions (PAFs). Previous studies have estimated PAFs for cancer incidence and sometimes mortality, but few studies have provided an analysis incorporating years of life lost (YLLs). YLLs provide a combined indicator which captures the frequency of death and the impact of premature deaths at ages below what is expected of the population.^4^ Applying cancer PAFs to measures of YLLs could therefore provide a more complete estimate of the impact that these different risk factors have on societies.

We aimed to estimate the burden of cancer deaths and YLLs in 2020 due to major cancer risk factors in seven countries selected for their range of wealth, health indicators, and health care systems, varied demographic and economic growth, and geographical location.^5^ The countries selected for this study included five countries considered as major emerging economies, also known as the BRICS nations (Brazil, Russia, India, China, and South Africa), as well as the United Kingdom (UK), and United States (US). Together, these seven countries represent more than half of the global burden of cancer deaths.^6^ This study will provide a comprehensive view of the potential impact of prevention of four risk factors (alcohol consumption, tobacco smoking, excess body weight, and human papillomavirus [HPV] infection) on cancer mortality in the seven countries in question, and discuss the findings in the local context of each country's cancer control and risk factor prevention policies. These risk factors were chosen due to their large attributable burden of cancers at the global level and their relationship with several cancer types per risk factor. The prevalence of these risk factors can also be relatively easy to measure in the population and could be reduced through population-level interventions.

## Methods

### Overall study design

In this international study of cancer mortality, we applied PAFs of alcohol consumption, tobacco smoking, excess body weight, and HPV to estimates of cancer deaths to calculate the number of potentially preventable cancer deaths that were attributable to the four major risk factors in Brazil, Russia, India, China, South Africa, the UK, and US. We then applied the number of preventable cancer deaths to estimates of the expected years of life remaining calculated from life tables to obtain the number of YLLs from preventable cancer deaths. Appendix p 5 summarises the workflow of the study.

### Risk factors and related cancers

The study assessed four major cancer risk factors contributing to attributable cancers worldwide and cancer types that have sufficient evidence of a causal link as classified by the International Agency for Research on Cancer (IARC) Monographs program and the IARC Handbooks of Cancer Prevention.^1^^,^^2^ We obtained PAFs of cancer incidence (alcohol consumption, excess body weight, and HPV) or mortality (tobacco smoking) and their 95% confidence intervals (CIs) specific to each country, sex, and age group from recently published global population-based studies.^7^, ^8^, ^9^, ^10^ We briefly describe the methods and key characteristics of each PAF study in Appendix p 2, and detailed descriptions of the methods are provided in each respective study.

Country-specific cancer deaths by 5-year age group (0–4, 5–9, …, 80–84, 85+) were extracted from the GLOBOCAN 2020 database for each cancer type related to the four risk factors and for all cancers combined, except non-melanoma skin cancer.^11^ The complete list of cancer types (defined using International Classification of Disease, tenth revision ICD-10) associated with each risk factor is listed in Appendix p 7.

### Potentially preventable cancer deaths

We calculated the number and 95% CIs of potentially preventable cancer deaths by multiplying the country-, sex-, and age-specific PAFs and their upper and lower 95% CIs for each cancer type with the corresponding number of cancer deaths in 2020 for people aged 25 years and over. We used the 1966 Segi-Doll world standard population, a common standard population used for global cancer estimates, to calculate age-standardised mortality rates (ASMR) and 95% CIs per 100,000 population for ages 25 and over.^12^

### Preventable years of life lost

We combined the age-specific number of potentially preventable cancer deaths and 95% CIs with the estimated years of life remaining in each country to obtain YLLs from cancer attributable to each risk factor. Life expectancy estimates by country and age group for 2019 were extracted from the World Health Organization (WHO) Global Health Observatory database (https://www.who.int/data/gho/data/indicators/indicator-details/GHO/gho-ghe-life-tables-by-country). A linear interpolation, as described by Aragon and colleagues,^13^ was used to calculate the expected years of life remaining for deaths between age interval *x* to *x + n*, where *x* represents age and *n* is the length of the age interval. In the study, *n* is equal to 5 years, except age group 85–99 years where *n* is 15 years. Age-standardised YLL rates (ASYR) and 95% CIs per 100,000 population were calculated as above. All analyses and data visualisations were carried out using Stata/IC version 17.0 (StataCorp, 2021. Stata Statistical Software: Release 17. College Station, TX: StataCorp LP).

In a sensitivity analysis, we used the WHO standard life table instead of the country-specific life tables to calculate YLLs in order to evaluate the impact of differences in life expectancies between countries on YLLs and ASYRs.^14^ The WHO standard life table is based on the highest projected life expectancy for the year 2050 which was 91.9 years for women in Japan and the Republic of Korea.^14^ In a further sensitivity analysis, we calculated the number of YLLs and ASYRs which from cancer death in the 30 to 69 age group i.e. the WHO definition of premature death. Results from our sensitivity analyses are described in Appendix p 3.

### Role of the funding source

The funder of the study had no role in study design, data collection, data analysis, data interpretation, writing of the manuscript, or decision to submit.

## Results

### Cancer mortality attributable to four risk factors

In Brazil, Russia, India, China, South Africa, the UK, and the US in 2020, an estimated 326,300 (187,400–467,900) cancer deaths and 5.9 million (3.3 million–8.6 million) YLLs were attributable to alcohol consumption. A total of 1.3 million (1.1 million–1.6 million) cancer deaths and 20.8 million (17.0 million–24.6 million) YLLs were attributable to tobacco smoking, 208,000 (163,300–254,800) cancer deaths and 3.1 million (2.4 million–3.8 million) YLLs were attributable to excess body weight, and 190,400 (183,100–200,300) cancer deaths and 4.0 million (3.9 million–4.2 million) YLLs were attributable to HPV infection. Together, the four risk factors caused 1.9 million (1.5 million–2.2 million) cancer deaths and 30.4 million (24.6 million–35.9 million) YLLs among the seven countries in 2020.

**Fig. 1:**
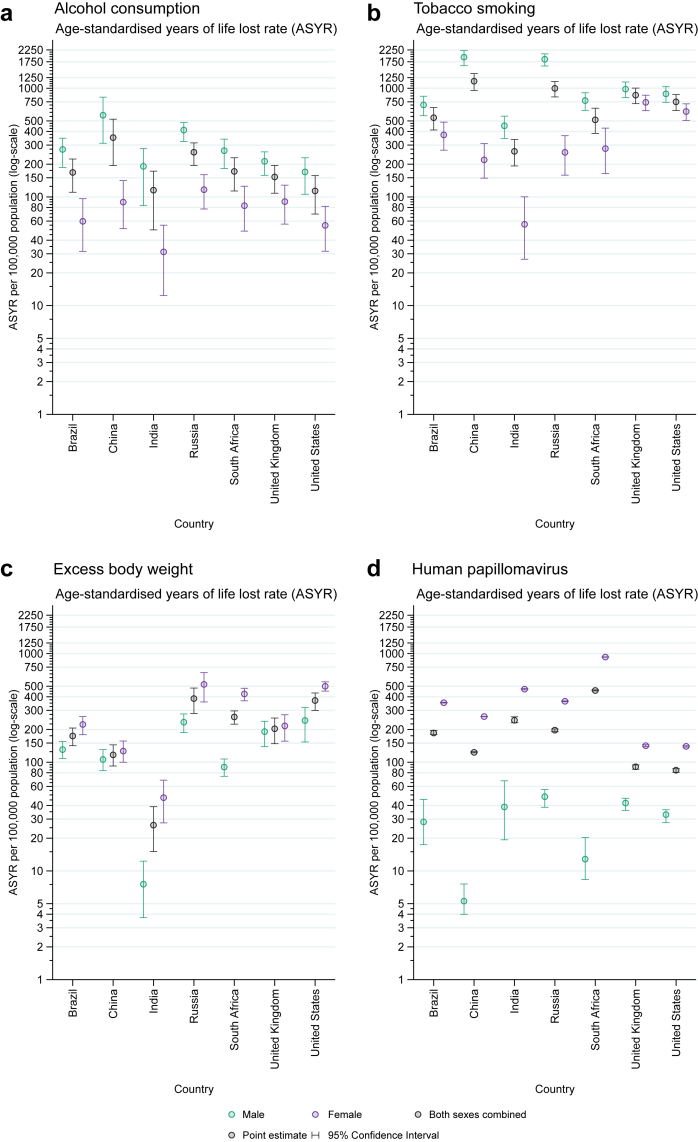
Age-standardised years of life lost rate (ASYR, per 100,000 population) from cancer deaths attributable to four risk factors: (a) alcohol consumption, (b) tobacco smoking, (c) excess body weight, (d) human papillomavirus by country and sex in 2020.

According to country, the ASYR due to alcohol consumption was highest in China (351.4 [194.5–519.2] YLLs per 100,000) and lowest in the US (113.5 [69.6–157.1]) and India (115.4 [49.7–172.7) (Fig. 1, Appendix p 8). China (1159.9 [950.6–1361.8]) had the highest ASYR from all cancers attributed to tobacco smoking, followed by Russia (996.8 [831.0–1154.5]), while India had the lowest (262.7 [192.7–337.5]). Russia (385.1 [280.6–481.2]) and the US (369.4 [299.6–433.6]) ranked the highest in ASYR attributable to excess body weight. For cancer deaths due to HPV infection, ASYRs ranged from 84.5 (80.8–87.2) in the US to 457.1 (453.3–462.6) in South Africa. ASMRs for each risk factor are presented in Appendix pp 6, 8.

### Sex differences in cancer mortality attributable to four risk factors

Overall, for both alcohol consumption and tobacco smoking, men had higher ASYRs in each country compared to women (Fig. 1). China, India, and Russia had the largest sex differences in ASYR due to alcohol-related cancers and tobacco smoking, with ASYRs among men up to nine times higher than those among women. In contrast to alcohol consumption and tobacco smoking, women had higher ASYRs for excess body weight and HPV infection compared to men across all countries in our study. ASYRs for excess body weight were six times higher among women in India and five times higher among women in South Africa than their male counterparts. Large sex differences were found for ASYRs due to HPV: up to 72 times higher among women than among men in China, 50 times higher among women than among men in South Africa, 12 times higher in Brazil and India, and between four and seven times higher in Russia, the UK, and US.

### Cancer mortality attributable to four risk factors by cancer type

**Fig. 2:**
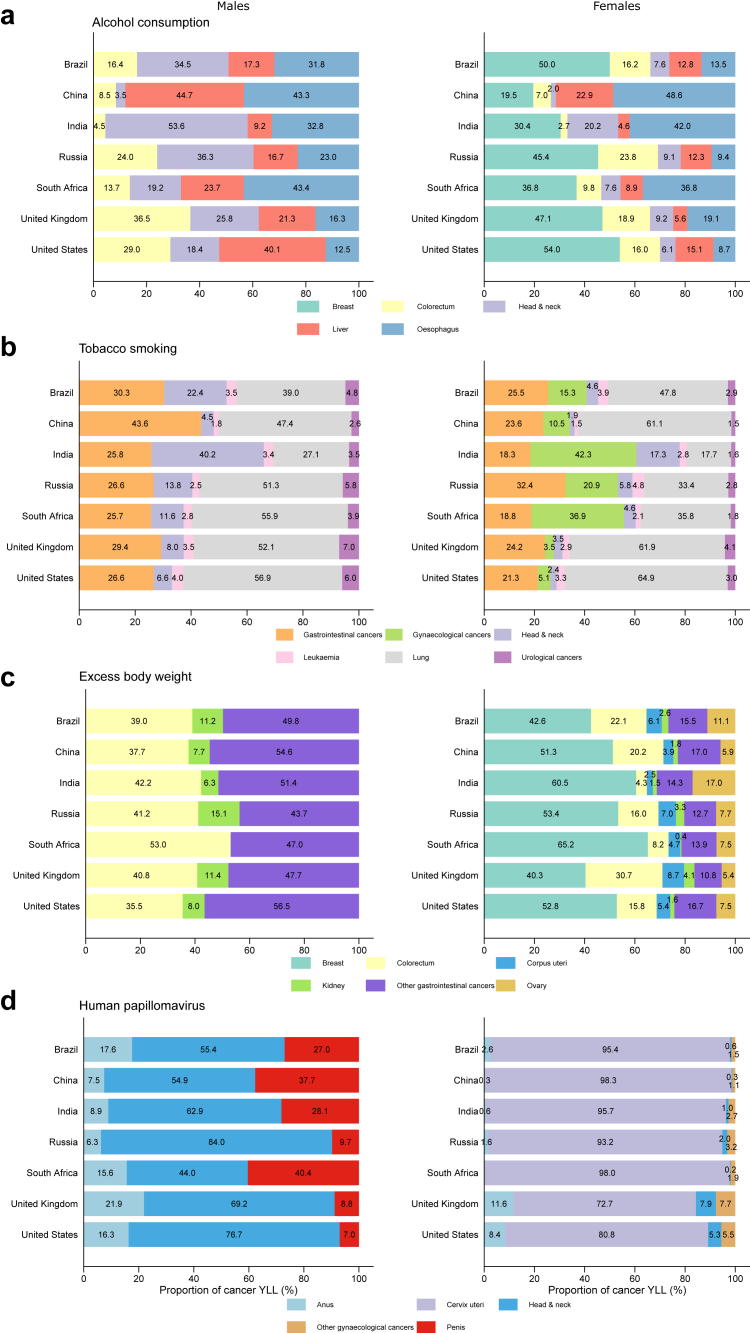
Proportion of years of life lost from cancer deaths attributable to four risk factors: (a) alcohol consumption, (b) tobacco smoking, (c) excess body weight, (d) human papillomavirus by sex, cancer type, and country in 2020.

The cancer types contributing the highest proportion of YLLs from cancer due to alcohol consumption varied between countries and between men and women (Fig. 2). Liver cancer contributed the most YLLs among men in China (44.7%, 1.5 million of 3.3 million) and the US (40.1%, 93,300 of 232,700) whereas colorectal cancer contributed the largest proportion in the UK (36.5%, 24,400 of 67,000). Head and neck cancers contributed more than half of YLLs from cancer due to alcohol among men in India (53.6%, 376,100 of 701,400) and more than a third in Brazil (34.5%, 60,100 of 174,200) and Russia (36.3, 77,700 of 214,100). Oesophageal cancer also contributed over 40% of YLLs due to alcohol among men in China (43.3%, 1.4 million of 3.3 million) and South Africa (43.4%, 14,000 of 32,400). Oesophageal cancer was also the major contributor of alcohol YLLs among women in China (48.6%, 259,900 of 534,900), South Africa (36.8%, 4600 of 12,500), and India (42.0%, 46,900 of 111,600), whereas breast cancer was the most important contributor to alcohol-related YLLs among women in Brazil (50.0%, 21,700 of 43,300), Russia (45.4%, 37,300 of 82,200), the UK (47.1%, 13,800 of 29,300), and US (54.0%, 41,600 of 77,000).

Lung cancer contributed the largest proportion of smoking-related YLLs in all countries and among both sexes except India where head and neck cancers were the main cause among men (40.2%, 639,300 of 1.6 million) and gynaecological cancers were the main driver among women (42.3%, 82,400 of 194,800), and South Africa where gynaecological cancers were also the main driver among women (36.9%, 15,200 of 41,200). For men, colorectal cancer and other gastrointestinal cancers contributed the most YLLs due to excess body weight. Breast cancer contributed the largest proportion of YLLs from excess body weight among women in all seven countries. Head and neck cancer contributed the most HPV-related YLLs among men in all seven countries, ranging from 44.0% (680 of 1700) in South Africa to 84.0% (18,000 of 24,600) in Russia. For all countries, cervical cancer contributed the large majority of YLLs from HPV among women, ranging between 72.7% (26,100 of 35,900) in the UK and 98.3% (1.4 million of 1.5 million) in China.

## Discussion

In this population-based study we examined the impact of four major cancer risk factors on deaths and YLLs from cancer. In Brazil, Russia, India, China, South Africa, the UK, and the US in 2020, an estimated 5.9 million cancer YLLs were attributable to alcohol consumption, 20.8 million YLLs were attributable to tobacco smoking, 3.1 million YLLs were attributable to excess body weight, and 4.0 million YLLs were attributable to HPV infection. ASYRs due to alcohol consumption and tobacco smoking were highest in China and Russia, and lowest in India. Russia and the US had the highest ASYRs attributable to excess body weight and South Africa had the highest HPV-attributable ASYRs. Men had higher ASYRs from cancer attributable to tobacco smoking and alcohol consumption than women, whereas women's ASYRs attributable to excess body weight and HPV infection were considerably higher than those among men. The distribution of YLLs by cancer type varied between men and women and between countries for alcohol consumption, highlighting differences in the impact of premature death from cancer attributable to the four risk factors.

Tobacco smoking contributed the most cancer deaths and YLLs in all seven countries, highlighting its major impact on cancer mortality. The prevalence of tobacco smoking in the UK and US peaked in the 1950s when around 50% of the adult population smoked; this dropped to 13% in the UK and the US in 2020–2021 largely thanks to tobacco control efforts,^15^^,^^16^ but the historically high smoking rates are still a driving factor of the cancer burden today. In China and Russia, where ASYRs attributable to tobacco smoking were highest, around half of men currently smoke.^17^ In China, stricter policies including higher excise taxes and restrictions on smoking in indoor public places are needed to meet the nation's Healthy China 2030 objective of reducing smoking prevalence to 20% by 2030.^18^ Meanwhile in Russia, ratification of the Framework Convention on Tobacco Control in 2008 has had a modest effect on tobacco prevalence among men and a subsequent small decline in male mortality, yet smoking prevalence among women is increasing and has surpassed that of the UK and the US, as reported in recent surveys.^19^ Thus, particularly in Russia, implementation of tobacco policies should consider gender and generational differences in smoking trends. In Brazil, tobacco smoking prevalence decreased from 35% in 1989 to 13% in 2019 due to successful control policies including advertising bans, smoke-free laws, and increased taxes.^20^ A decline in smoking prevalence in India has also been observed, which could be due to strong tobacco control regulations that have been put in place since the launch of the National Tobacco Control Plan in 2007–08; these regulations have focused on national public awareness campaigns, increased prominence of health warnings on packaging, and expanded cessation facilities, for both smoking and smokeless forms of tobacco.^21^

After tobacco smoking, cancer attributable to HPV infection was a major contributor to YLLs in South Africa and India. The elevated ASYRs in South Africa and India could have been due to suboptimal access to cervical cancer screening and treatment,^22^^,^^23^ which could also be the case among the other BRICS nations. Although we found a relatively low ASYR due to HPV in Brazil, mortality rates are very unequal between regions and extremely high rates of premature mortality are found in the North of Brazil.^24^ Within Brazil, there are regional inequalities in HPV vaccination rates and coverage of cervical cancer screening which remains opportunistic.^25^^,^^26^ China provides an example of an ambitious yet achievable cervical cancer elimination plan of initiatives aimed at boosting HPV vaccination rates among girls, stepping up screening efforts among women to screen at least 70% of eligible women, and to treat 90% of patients with cervical cancer and precancerous lesions by 2030.^27^

Alcohol consumption has also contributed to the elevated ASYRs we found in China, Russia, and the UK. Alcohol use in Russia and the UK has decreased over recent decades but alcohol control efforts have recently stalled,^28^ and alcohol consumption is projected to grow in Russia by 2025.^29^ Alcohol consumption is also predicted to increase in China unless policies to reduce population alcohol use such as increases in excise taxes, restrictions on marketing, and limiting the availability of alcohol products are implemented at the highest levels.^30^ Moreover, although a significant part of the population in Brazil abstains from drinking, prevalence of heavy drinking is on the rise.^31^ Excess body weight was also an important contributor to ASYRs in Russia, the US, and the UK, which is largely due to the growing obesity epidemic that has accompanied the economic growth and urbanisation of each country.^32^ Overweight and obesity prevalence in Russia is now comparable to the US and is higher than other European countries.^33^ Furthermore, the impact of excess body weight on cancer burden in Brazil is likely to increase further as the prevalence of overweight and obesity has grown significantly in recent years, from 57.0% in 2013 to 60.3% in 2019.^34^

We found stark differences in the rate of preventable YLLs between men and women in each country which varied according to risk factor. Generally, men had a higher burden of YLLs attributable to alcohol use and tobacco smoking in all seven countries, which is largely linked to higher consumption among men.^17^^,^^29^ This variation was much smaller in the UK and US where alcohol consumption and tobacco smoking among women have increased to similar rates to those of men.^17^^,^^29^ In contrast, female ASYRs attributable to excess body weight and HPV infection were higher than their male counterparts. Sex-differences in YLLs due to excess body weight could be explained by higher prevalence of excess body weight among women than men, and a larger number of female-specific cancers associated with excess body weight than male cancers.^9^ This was also the case for HPV-attributable YLLs where female cancers, cervical cancer in particular, were the main contributors. For these four risk factors, cultural norms might have influenced differences in exposure due to how men and women's roles in societies are perceived, including more social activities among men and stigma towards women's use of substances associated with the male identity. Exposure has been further impacted by commercial determinants such as the tobacco, alcohol, and food industries which have targeted gender roles to increase sales and consumption in many economically developed countries, and increasingly in low- and middle-income countries.^35^ Cancer prevention policies should counteract these societal and commercial influences to reduce inequalities in deaths and YLLs between men and women.

Other studies such as the Global Burden of Disease (GBD) study have also estimated YLLs due to cancer attributable to major risk factors as part of their estimation of disease burden.^8^ Using the WHO world standard life table, GBD reported 32.6 million cancer YLLs due to tobacco smoking, 7.2 million due to alcohol consumption, 5.6 million due to high BMI, and 4.0 million due to unsafe sex in the seven countries in our study in 2019. Our estimates using the WHO world standard were generally comparable to those of GBD but differences at the country and cancer type level stemmed from several sources. This was particularly evident for lung cancer in the United States where GBD reported a 51% higher crude rate of lung cancer death than the rate we obtained from GLOBOCAN (63.0 versus 41.8 deaths per 100,000). Such discrepancies in the underlying number of cancer deaths reported by GBD and GLOBOCAN are due to the different modelling methods employed by each study to estimate cancer burden. The GBD study used a global approach to model patterns of disease based on data from mortality registries as well as the prevalence of cancer risk factors or pre-cancers to impute missing cancer data. The GLOBOCAN developers used a data-based approach which prioritised locally collected cancer incidence and mortality data. In addition to discrepancies in the underlying rates of cancer death, our YLL results differed from those of the GBD due to the PAFs we used which were based on other sources of prevalence and relative risks. The GBD study also only considered the relationship between HPV and cervical cancer and did not include cancers at other gynaecological sites, penile cancer, anal squamous cell carcinoma, nor cancers of the oral cavity or oropharynx, and so underestimated YLLs due to HPV infection. Another global study estimated the avoidable burden of cancer deaths and YLLs by considering the impact of YLLs from premature death that were preventable or treatable.^36^ This study assumed that all premature deaths (age 30–69) are avoidable and used criteria based on PAFs and five-year net survival of each cancer type to distinguish between deaths that were avoidable through primary or secondary prevention and those that were avoidable through improved treatment with curative intent. Frick et al. estimated that 72.9 million YLLs in the BRICS countries, UK, and US were avoidable through prevention, which is around twice our estimate of 36.4 million premature YLLs because our study used PAFs to estimate preventable deaths and YLLs whether Frick et al. considered all deaths from a theoretically ‘preventable’ cancer type as preventable, therefore providing an ultimate scenario where all premature deaths are avoidable.

The strengths of our study include the quantification of cancer YLLs using PAFs from four global population-based studies on alcohol consumption, tobacco smoking, excess body weight, and HPV infection. By presenting YLLs we have provided a composite estimate of both the frequency of cancer death and the impact of premature cancer death which is powerful in setting priorities to improve public health. Furthermore, the four PAF studies each used comparable and consistent methods and prevalence data across the BRICS countries, the UK, and US. Despite this, a limitation to our study is that we only included four major modifiable cancer risk factors while studies have shown the impact of other major risk factors such as *Helicobacter pylori*, hepatitis B and C virus infection, occupational exposures, and air pollution.^37^^,^^38^ Chewing tobacco is also a key risk factor for cancer in India and other South Asian countries where consumption is common.^39^ Further YLL studies could incorporate additional emerging risk factors including air pollution which evidence has suggested is a driver in the evolving epidemiology of lung cancer,^40^ and processed meat consumption and physical inactivity in the rising incidence of colorectal cancer among younger adults.^41^

Another limitation to our study is that we did not include the potential synergistic effect between the four risk factors and cancer, such as that between alcohol and tobacco use which is particularly relevant for cancers of the upper aerodigestive tract.^7^ Population-based PAF studies usually try to combat potential confounding by using relative risks adjusted for other risk factors, sex, and age. When these are derived from meta-analyses of observational studies reporting risk estimates adjusted inconsistently from one study to the next, as was the case for the PAFs we used for alcohol consumption, tobacco smoking, and excess body weight, unmeasured confounding remains an important limitation. An alternative method of computing PAFs which can potentially remove confounding by related risk factors is Miettinen's formula which uses risk factor prevalence among cases or deaths.^42^ The studies which calculated PAFs of alcohol consumption, tobacco smoking, and excess body weight did not have access to reports of prevalence among cancer cases and instead based their calculations on the method which uses population prevalence; they therefore did not eliminate bias due to confounding which has thus been carried through to our estimates of deaths attributable to the relevant risk factors. It is also important to note that we used PAFs relative to a theoretical situation where no one in the population was exposed to the risk factors; while this is an optimistic outlook, a comparison modelling reduction in exposure through specific interventions might provide a more realistic goal which is perhaps more achievable in today's society. In addition, we assumed that PAFs of cancer incidence attributable to alcohol consumption, excess body weight, and HPV infection were applicable to cancer mortality. Other PAF studies which have incorporated both PAFs of cases and deaths have used relative risks for cancer incidence applied to mortality due to a larger number of high-quality relative risk studies for cancer incidence than mortality.^38^ This assumes that the risk relationship due to these risk factors is the same for cancer incidence as for cancer mortality; but exposure to these risk factors might worsen outcomes for patients diagnosed which is particularly evident for specific cancer subtypes which have poorer survival than other subtypes such as oesophageal squamous cell carcinoma compared with adenocarcinoma.^43^ Similarly, we used general population life tables to calculate the expected years of life remaining at cancer death, but this did not account for the potentially lower life expectancy among people exposed to each risk factor and a higher risk of mortality from comorbidities related to those risk factors. Finally, when we used the WHO standard life table as a reference to calculate YLLs, we estimated many more YLLs than in the main analysis. WHO's standard life expectancy was higher than the seven countries in our study because it was based on the longest projected life expectancies for the year 2050.

In conclusion, alcohol consumption, tobacco smoking, excess body weight and HPV infection caused a substantial burden of cancer mortality and YLLs in Brazil, China, India, Russia, South Africa, the UK, and US in 2020. While tobacco smoking was the predominant risk factor among all seven countries, the remaining risk factors varied in their contributions to cancer mortality, which largely reflect exposure in the population and differences in the mortality of risk factor-related cancers in each country. Our findings demonstrate the importance of cancer control efforts to reduce the burden of cancer death and YLLs due to modifiable cancer risk factors and the usefulness of YLLs to summarise disease burden.

## Contributors

PS acquired funding for the study; CJC, IS, JO, PS conceived the study; CJC, HR contributed to study design and analysis; CJC, HR accessed, collected, and verified the underlying data; CJC, HR, IS, JO, PS interpretation of the results; CJC, HR wrote the first draft of the manuscript. All authors critically reviewed the manuscript and agreed with the decision to submit for publication.

## Data sharing statement

The cancer mortality data used in this study are retrievable from the publicly available platform, the Global Cancer Observatory (GLOBOCAN) https://gco.iarc.fr/. The cancer PAFs for alcohol, infections, and excess body weight are also publicly available via the Global Cancer Observatory. The cancer PAFs for tobacco smoking are available on the GBD Results tool https://vizhub.healthdata.org/gbd-results/. The life expectancy estimates are also publicly available via the WHO's Global Health Observatory https://bit.ly/37va2Gf. All statistical code (ie, Stata code) and input files used to produce the results presented in this paper are available to the public on request to the corresponding author.

## Declaration of interests

The authors have no conflicts of interest to declare. Where authors are identified as personnel of the International Agency for Research on Cancer/World Health Organization, the authors alone are responsible for the views expressed in this article and they do not necessarily represent the decisions, policy or views of the International Agency for Research on Cancer/World Health Organization.

## References

[1] International Agency for Research on Cancer (2023). https://publications.iarc.fr/Book-And-Report-Series/Iarc-Monographs-On-The-Identification-Of-Carcinogenic-Hazards-To-Humans.

[2] International Agency for Research on Cancer (2018). https://publications.iarc.fr/570.

[3] World Cancer Research Fund/American Institute for Cancer Research (2018). Diet, nutrition, physical activity and cancer: a global perspective. Continuous update project expert report. https://www.wcrf.org/wp-content/uploads/2021/02/Summary-of-Third-Expert-Report-2018.pdf.

[4] Martinez R., Soliz P., Caixeta R., Ordunez P. (2019). Reflection on modern methods: years of life lost due to premature mortality—a versatile and comprehensive measure for monitoring non-communicable disease mortality. Int J Epidemiol.

[5] Pearce A., Sharp L., Hanly P. (2018). Productivity losses due to premature mortality from cancer in Brazil, Russia, India, China, and South Africa (BRICS): a population-based comparison. Cancer Epidemiol.

[6] Ferlay J., Ervik M., Lam F. (2021). https://gco.iarc.fr/today/home.

[7] Rumgay H., Shield K., Charvat H. (2021). Global burden of cancer in 2020 attributable to alcohol consumption: a population-based study. Lancet Oncol.

[8] Tran K.B., Lang J.J., Compton K. (2022). The global burden of cancer attributable to risk factors, 2010-19: a systematic analysis for the Global Burden of Disease Study 2019. Lancet.

[9] Arnold M., Pandeya N., Byrnes G. (2015). Global burden of cancer attributable to high body-mass index in 2012: a population-based study. Lancet Oncol.

[10] de Martel C., Georges D., Bray F., Ferlay J., Clifford G.M. (2020). Global burden of cancer attributable to infections in 2018: a worldwide incidence analysis. Lancet Glob Health.

[11] Ferlay J., Colombet M., Soerjomataram I. (2021). Cancer statistics for the year 2020: an overview. Int J Cancer.

[12] Doll R., Payne P., Waterhouse J. (1966). https://link.springer.com/book/9783540034759.

[13] Aragon T.J., Lichtensztajn D.Y., Katcher B.S., Reiter R., Katz M.H. (2008). Calculating expected years of life lost for assessing local ethnic disparities in causes of premature death. BMC Public Health.

[14] World Health Organization (2017).

[15] Office for National Statistics (2021).

[16] Office on Smoking and Health NCfCDPaHP (2020).

[17] World Health Organization (2021).

[18] Li X., Galea G. (2019). Healthy China 2030: an opportunity for tobacco control. Lancet.

[19] Shkolnikov V.M., Churilova E., Jdanov D.A. (2020). Time trends in smoking in Russia in the light of recent tobacco control measures: synthesis of evidence from multiple sources. BMC Public Health.

[20] Tam J., Jaffri M.A., Mok Y. (2023). Patterns of birth cohort‒specific smoking histories in Brazil. Am J Prev Med.

[21] Tata Institute of Social Sciences (TISS) & Ministry of Health and Family Welfare Government of India (2017).

[22] Bhatla N., Meena J., Kumari S., Banerjee D., Singh P., Natarajan J. (2021). Cervical cancer prevention efforts in India. Indian J Gynecol Oncol.

[23] Union for International Cancer Control (UICC) (2022).

[24] De Camargo Cancela M., Bezerra de Souza D.L., Leite Martins L.F. (2023). Can the sustainable development goals for cancer be met in Brazil? A population-based study. Front Oncol.

[25] Moura LdL., Codeço C.T., Luz P.M. (2021). Cobertura da vacina papilomavírus humano (HPV) no Brasil: heterogeneidade espacial e entre coortes etárias. Rev Bras Epidemiol.

[26] Costa R.F.A., Longatto-Filho A., de Lima Vazquez F., Pinheiro C., Zeferino L.C., Fregnani J.H.T.G. (2018). Trend analysis of the quality indicators for the Brazilian cervical cancer screening programme by region and state from 2006 to 2013. BMC Cancer.

[27] National Health Commission of the People’s Republic of China Notice on issuing the action plan for accelerating the elimination of cervical cancer (2023-2030). http://www.nhc.gov.cn/fys/s3581/202301/42c2c95b6db84f9cb356cfdf1edbbac7.shtml.%20Date.

[28] WHO Regional Office for Europe (2016).

[29] World Health Organization (2018).

[30] Manthey J., Shield K.D., Rylett M., Hasan O.S.M., Probst C., Rehm J. (2019). Global alcohol exposure between 1990 and 2017 and forecasts until 2030: a modelling study. Lancet.

[31] Silva L.E.S.D., Helman B., Luz e Silva D.C.D. (2022). Prevalência de consumo abusivo de bebidas alcoólicas na população adulta brasileira: pesquisa Nacional de Saúde 2013 e 2019. Epidemiolo Serv Saúde.

[32] Malik V.S., Willet W.C., Hu F.B. (2020). Nearly a decade on — trends, risk factors and policy implications in global obesity. Nat Rev Endocrinol.

[33] Kontsevaya A., Shalnova S., Deev A. (2019). Overweight and obesity in the Russian population: prevalence in adults and association with socioeconomic parameters and cardiovascular risk factors. Obes Facts.

[34] Ferreira A.P.S., Szwarcwald C.L., Damacena G.N., Souza Júnior P.R.B. (2021). Increasing trends in obesity prevalence from 2013 to 2019 and associated factors in Brazil. Rev Bras Epidemiol.

[35] Feeny E., Dain K., Varghese C., Atiim G.A., Rekve D., Gouda H.N. (2021). Protecting women and girls from tobacco and alcohol promotion. BMJ.

[36] Frick C., Rumgay H., Vignat J. (2023). Quantitative estimates of preventable and treatable deaths from 36 cancers worldwide: a population-based study. Lancet Glob Health.

[37] Brown K.F., Rumgay H., Dunlop C. (2018). The fraction of cancer attributable to modifiable risk factors in England, Wales, Scotland, Northern Ireland, and the United Kingdom in 2015. Br J Cancer.

[38] Islami F., Goding Sauer A., Miller K.D. (2018). Proportion and number of cancer cases and deaths attributable to potentially modifiable risk factors in the United States. CA Cancer J Clin.

[39] Siddiqi K., Husain S., Vidyasagaran A., Readshaw A., Mishu M.P., Sheikh A. (2020). Global burden of disease due to smokeless tobacco consumption in adults: an updated analysis of data from 127 countries. BMC Med.

[40] Lortet-Tieulent J., Soerjomataram I., Ferlay J., Rutherford M., Weiderpass E., Bray F. (2014). International trends in lung cancer incidence by histological subtype: adenocarcinoma stabilizing in men but still increasing in women. Lung Cancer.

[41] Araghi M., Soerjomataram I., Bardot A. (2019). Changes in colorectal cancer incidence in seven high-income countries: a population-based study. Lancet Gastroenterol Hepatol.

[42] Mansournia M.A., Altman D.G. (2018). Population attributable fraction. BMJ.

[43] Morgan E., Soerjomataram I., Gavin A.T. (2021). International trends in oesophageal cancer survival by histological subtype between 1995 and 2014. Gut.

