# Supplementary material for: International burden of cancer deaths and years of life lost from cancer attributable to four major risk factors: a population-based study in Brazil, Russia, India, China, South Africa, the United Kingdom, and United States
Source: eClinicalMedicine. 2023 Nov 15;66:102289. doi: 10.1016/j.eclinm.2023.102289 (PMC10772154; doi:10.1016/j.eclinm.2023.102289)
Supplement: Appendix [file mmc1.docx]

**Supplementary appendix**

**Contents**

Supplemental methods2

Description of population attributable fractions methods2

Estimation of cancer mortality for histological subtypes or topographical subsites2

Sensitivity analyses3

Using WHO standard life table to calculate years of life lost3

Calculating years of life lost between ages 30 and 693

Appendix figure 1. Workflow of the study design, data sources, and methods used in the present study5

Appendix figure 2. Age-standardised mortality rate (ASMR, per 100,000 population) from cancer deaths attributable to four risk factors: (a) alcohol consumption, (b) tobacco smoking, (c) excess body weight, (d) human papillomavirus by country and sex in 2020.6

Appendix table 1. Selection of cancer types associated with alcohol consumption, tobacco smoking, excess body weight, and human papillomavirus7

Appendix table 2. Age-standardised rates of mortality and years of life lost from preventable cancer deaths in Brazil, China, India, Russia, South Africa, the United Kingdom, and the United States in 2020 due to alcohol consumption, tobacco smoking, excess body weight, and human papillomavirus8

Appendix table 3. Number of preventable cancer deaths and years of life lost in Brazil, China, India, Russia, South Africa, the United Kingdom, and the United States in 2020 due to four major risk factors9

Appendix table 4. Number and age-standardised rates of years of life lost from preventable cancer deaths in Brazil, China, India, Russia, South Africa, the United Kingdom, and the United States in 2020 due to four major risk factors using the WHO standard life table (sensitivity analysis), by sex 11

Appendix table 5. Number and age-standardised rates of years of life lost from preventable cancer deaths occurring between ages 30 and 69 in Brazil, China, India, Russia, South Africa, the United Kingdom, and the United States in 2020 due to four major risk factors using the WHO standard life table (sensitivity analysis), by sex 12

References.23

**Supplemental methods**

*Description of population attributable fractions methods*

We extracted estimates of population attributable fractions (PAFs) of cancer incidence (alcohol consumption, excess body weight, and HPV) or cancer mortality (tobacco smoking) and their 95% confidence intervals (CIs) specific to each country, sex, and age group from recently published global population-based studies.^1-4^ The PAFs of alcohol consumption, tobacco smoking, and excess body weight were calculated following a Levin-based PAF formula whereby the relative risk of cancer from each risk factor was combined with the sex and age-specific prevalence of risk factor exposure in the population. Relative risks adjusted for confounding factors were obtained from meta-analyses.

In the case of alcohol consumption, tobacco smoking, and excess body weight, the theoretical minimum risk for PAF was estimated as no exposure i.e. lifetime abstention from alcohol, never smoking, or body mass index 22 kg/m^2^. Tobacco smoking PAFs were estimated based on modelled current pack-years of smoking;^5^ alcohol consumption PAFs used g ethanol per day; and excess body weight PAFs measured body mass index > 22 kg/m^2^. Alcohol and excess body weight PAFs factored in a 10-year latency period between prevalence and related cancer incidence based on an average follow-up time of 10 years in epidemiological studies from which the relative risks used in the PAF calculations were derived.^1,3^ To calculate the PAFs due to HPV, de Martel et al. assumed cervical cancer and squamous cell carcinoma of the anus were 100% attributable to HPV infection and obtained the prevalence of HPV infection in cancer cases from published reports for other cancer sites related to HPV.^4^

*Estimation of cancer mortality for histological subtypes or topographical subsites*

Due to specific causal associations between the risk factors and specific histological subtypes or topographical sites of some cancer types, estimates of cancer deaths by subtype or subsite were used in the preventable cancer deaths analysis for the relevant risk factor-cancer type combinations. This was the case for: alcohol consumption and oesophageal squamous cell carcinoma and hepatocellular carcinoma; excess body weight and oesophageal adenocarcinoma, cardia stomach cancer, and hepatocellular carcinoma; and HPV and oral cavity cancer, oropharyngeal cancer, and anal squamous cell carcinoma.

The data sources and detailed methods in compiling the estimates for each histological subtype and subsite have been described elsewhere.^4,6,7^ Briefly, the proportions of each subtype and subsite out of the total number of cases of their overarching cancer type by sex, country, and age were calculated using cancer registry data from Cancer Incidence in Five Continents (CI5) volume XI.^8^ The relevant histological subtypes or topographical subsites were defined using the International Classification of Diseases for Oncology, 3rd edition (ICD-O-3) for oesophageal adenocarcinoma (morphology codes 8140–8141, 8143–8145, 8190–8231, 8260–8265, 8310, 8401, 8480–8490, 8550–8552, 8570–8574, 8576), oesophageal squamous cell carcinoma (8050-8078, 8083-8084), hepatocellular carcinoma (8170-8175), and anal squamous cell carcinoma (8050–8076, 8083–8084, 8123-8124); and International Classification of Disease, tenth revision (ICD-10) for cardia stomach cancer (ICD-10 C16.0).

We applied similar methodology to that described above to obtain the number of cancer deaths from cancer of the oropharynx (C01, C09-10), oral cavity only (C02-06), and kidney excluding renal pelvis (C64) which were grouped with other sites in the GLOBOCAN 2020 estimates, as detailed in appendix p 7.

**Sensitivity analyses**

*Using WHO standard life table to calculate years of life lost*

In our sensitivity analysis we used the WHO standard life table to calculate YLLs in each country. There were more YLLs and higher ASYRs across all four risk factors in all seven countries when using the WHO standard life table compared with the estimates from the main analysis which used country-specific life tables (appendix p 10). Differences between the estimates using the WHO standard life table and the country-specific tables were smallest in the UK and US and highest among the BRICS countries, but the overall ranking of ASYRs among the seven countries remained the same as the main analysis i.e. highest in China and Russia, lowest in India and Brazil. The ASYRs due to all four risk factors combined were 41% higher in the UK (1,640·7 [95% CI 1,365·6–1,895·7]) and 45% higher in the US (1,606·5 [1,346·8–1,847·9]) when we used the WHO standard life table versus the country-specific life tables. The ASYR using the WHO standard life table was 51% higher for Brazil and 60% higher for China than when using their respective life tables. The ASYR was 67% higher for Russia (2,560·4 [2,164·6–2,907·7]), 70% higher for India (991·7 [773·1–1,204·1]), and 73% higher for South Africa (2,065·9 [1,773·0–2,365·3]).

*Calculating years of life lost between ages 30 and 69*

When we calculated YLLs of deaths between ages 30 and 69 (premature mortality), we estimated YLLs among men ranging from 189,400 deaths per 100,000 (149,100–225,500) in South Africa to 18,159,200 (14,511,100–21,329,800) in China (appendix p12). YLLs occurring prematurely among women were also lowest in South Africa (356,900 [323,800–395,200]) and highest in China 5,582,400 (4,515,500–6,883,300). Patterns of ASYRs differed with the lowest ASYRs among men in India (1,133.3 [782.0–1,437.0]) and the highest in Russia (4,252.7 [3,695.8–4,719.2]). We found the lowest ASYRs among women in India (917.2 [826.4–1,034.7]) and China (925.7 [753.4–1,136.6]) and the highest in South Africa (2,293.0 [2,072.6–2,547.3]).

**
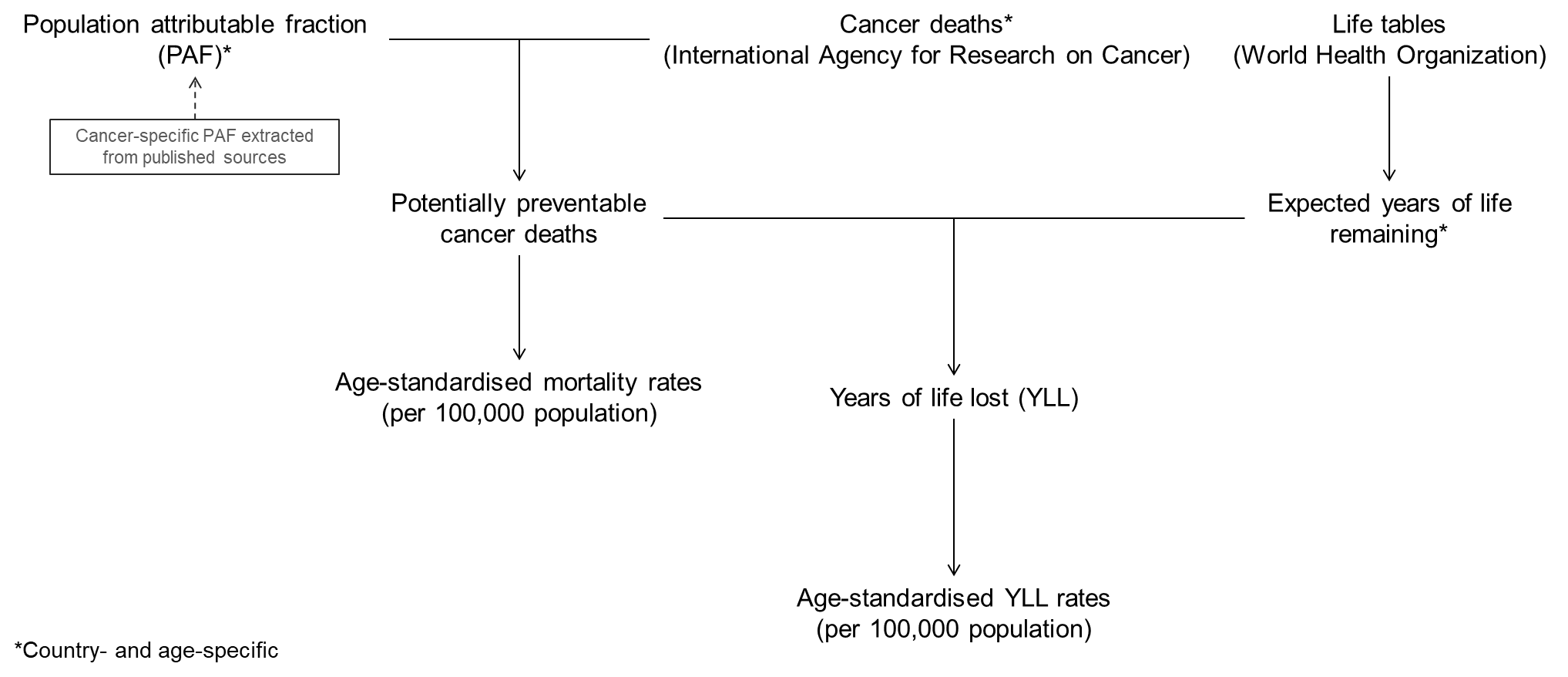
**

**Appendix figure 1**. Workflow of the study design, data sources, and methods used in the present study.

**
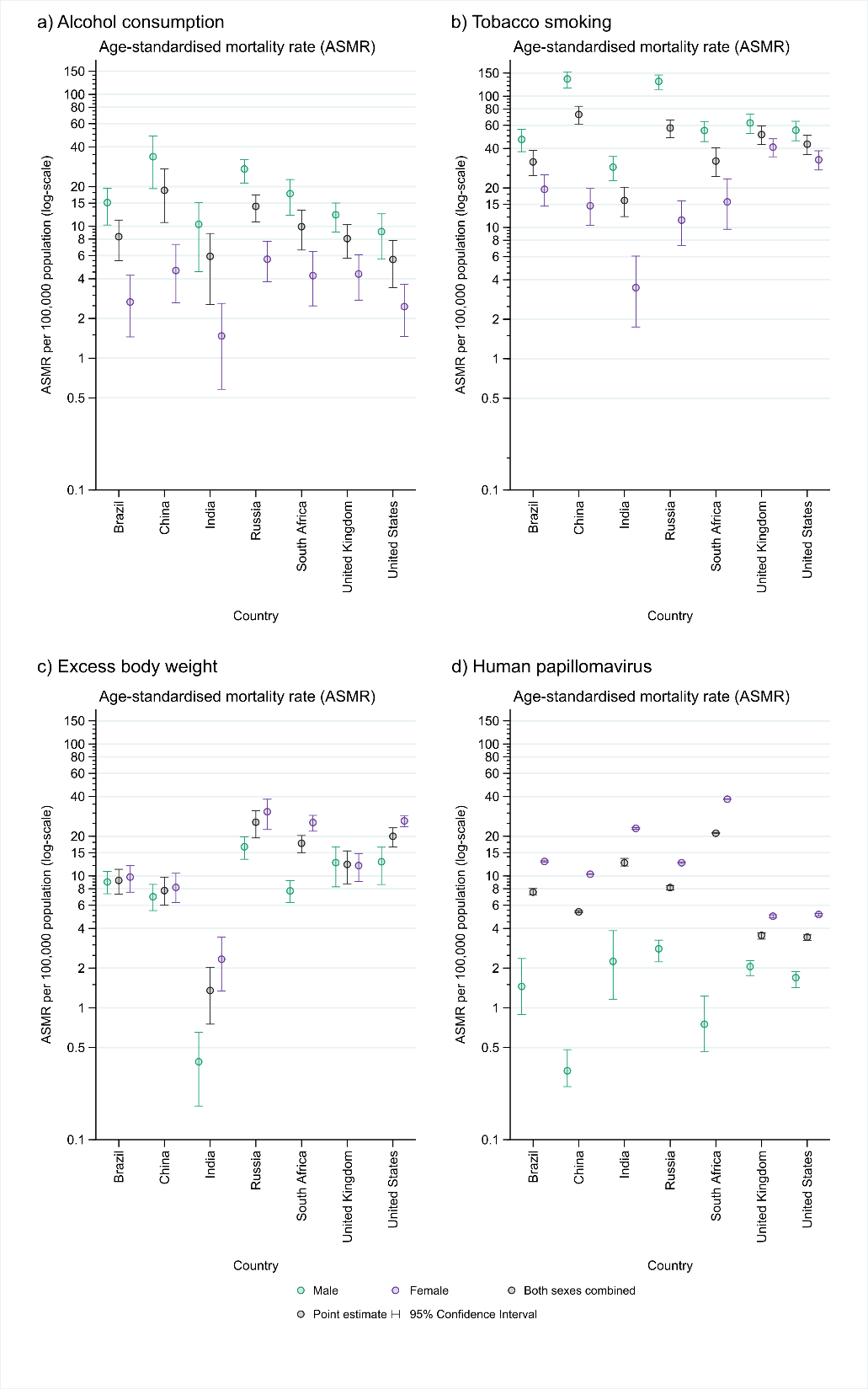
**

**Appendix figure 2**. Age-standardised mortality rate (ASMR, per 100,000 population) from cancer deaths attributable to four risk factors: (a) alcohol consumption, (b) tobacco smoking, (c) excess body weight, (d) human papillomavirus by country and sex in 2020.

**Appendix table 1**. Selection of cancer types associated with alcohol consumption, tobacco smoking, excess body weight, and human papillomavirus according to the sources of the population attributable fractions.

| **Cancer type** | **ICD-10 grouping** | **Alcohol consumption (Rumgay H et al. 2021)^1^** | **Tobacco smoking (GBD 2019)^2^** | **Excess body weight (Arnold M et al. 2015)^3^** | **Human papillomavirus (de Martel C et al. 2020)^4^** |
| --- | --- | --- | --- | --- | --- |
| Lip, oral cavity | C00-06 | X | X |  | X - Oral cavity (C02-C06) |
| Oropharynx | C09-10 | X | X |  | X - Oropharynx (C01, C09-10) |
| Nasopharynx | C11 |  | X |  |  |
| Hypopharynx | C12-13 | X | X |  |  |
| Oesophagus | C15 | X - Squamous cell carcinoma | X | X - Oesophageal adenocarcinoma |  |
| Stomach | C16 |  | X | X - Cardia |  |
| Colon | C18 | X | X | X |  |
| Rectum | C19-20 | X | X | X |  |
| Anus | C21 |  |  |  | X - Squamous cell carcinoma |
| Liver and intrahepatic bile ducts | C22 | X - Hepatocellular carcinoma | X | X - Hepatocellular carcinoma |  |
| Gallbladder | C23 |  |  | X |  |
| Pancreas | C25 |  | X | X |  |
| Larynx | C32 | X | X |  | X |
| Trachea, bronchus and lung | C33-34 |  | X |  |  |
| Breast | C50 | X |  | X - Postmenopausal |  |
| Vulva | C51 |  |  |  | X |
| Vagina | C52 |  |  |  | X |
| Cervix uteri | C53 |  | X |  | X |
| Corpus uteri | C54 |  |  | X |  |
| Ovary | C56 |  | X - Mucinous ovary* | X |  |
| Penis | C60 |  |  |  | X |
| Kidney and renal pelvis | C64-65 |  | X | X – Kidney only |  |
| Bladder | C67 |  | X |  |  |
| Leukaemia | C91-95 |  | X |  |  |

*PAF for mucinous ovary cancer calculated following Cao B et al. 2018.^9^ Abbreviations: ICD-10=International Classification of Disease, tenth revision.

**Appendix table 2.** Age-standardised rates of mortality and years of life lost from preventable cancer deaths in Brazil, China, India, Russia, South Africa, the United Kingdom, and the United States in 2020 due to alcohol consumption, tobacco smoking, excess body weight, and human papillomavirus.

|  | **Brazil** | | **China** | | **India** | | **Russia** | | **South Africa** | | **United Kingdom** | | **Unites States** | |
| --- | --- | --- | --- | --- | --- | --- | --- | --- | --- | --- | --- | --- | --- | --- |
|  | **ASMR** | **ASYR** | **ASMR** | **ASYR** | **ASMR** | **ASYR** | **ASMR** | **ASYR** | **ASMR** | **ASYR** | **ASMR** | **ASYR** | **ASMR** | **ASYR** |
| **Alcohol consumption** | 8·3 (5·5–11·1) | 168·0 (110·4–223·1) | 18·7 (10·7–27·3) | 351·4 (194·5–519·2) | 5·9 (2·6–8·8) | 115·4 (49·7–172·7) | 14·2 (10·8–17·3) | 257·6 (194·9–313·9) | 9·9 (6·6–13·3) | 171·7 (113·5–229·1) | 8·1 (5·7–10·3) | 152·8 (108·1–195·1) | 5·6 (3·4–7·8) | 113·5 (69·6–157·1) |
| **Tobacco smoking** | 31·6 (24·8–38·7) | 535·7 (412·6–664·0) | 72·5 (60·9–83·9) | 1159·9 (950·6–1361·8) | 16·1 (12·1–20·3) | 262·7 (192·7–337·5) | 57·3 (48·3–65·8) | 996·8 (831·0–1154·5) | 32·1 (24·5–40·4) | 511·9 (383·9–654·4) | 51·0 (42·7–59·4) | 863·2 (721·6–1002·8) | 43·1 (35·9–50·4) | 749·4 (624·0–874·9) |
| **Excess body weight** | 9·3 (7·3–11·2) | 174·5 (141·7–206·4) | 7·8 (6·0–9·8) | 116·9 (92·3–144·4) | 1·4 (0·8–2·0) | 26·4 (15·1–39·0) | 25·6 (19·4–31·3) | 385·1 (280·6–481·2) | 17·7 (15·0–20·3) | 260·8 (223·8–296·5) | 12·2 (8·7–15·5) | 203·1 (147·9–254·8) | 20·0 (16·6–23·2) | 369·4 (299·6–433·6) |
| **Human papillomavirus** | 7·5 (7·2–8·0) | 185·8 (179·3–195·9) | 5·3 (5·3–5·4) | 123·3 (122·2–124·9) | 12·6 (11·9–13·6) | 243·3 (230·4–261·7) | 8·2 (7·8–8·4) | 197·3 (190·9–202·3) | 21·1 (20·9–21·4) | 457·1 (453·3–462·6) | 3·5 (3·3–3·7) | 90·8 (86·1–94·4) | 3·4 (3·2–3·6) | 84·5 (80·8–87·2) |
| **All four risk factors combined** | 50.1 (40.4–59.6) | 933.1 (758.0–1,104.9) | 93.4 (76.3–109.8) | 1,560.9 (1,248.5–1,858.3) | 32.3 (25.4–39.1) | 582.6 (453.6–708.2) | 87.6 (74.4–99.3) | 1,533.7 (1,295.4–1,742.6) | 68.6 (58.3–79.1) | 1,197.6 (1,029.7–1,369.4) | 66.6 (55.2–77.3) | 1,160.2 (966.2–1,339.6) | 61.3 (51.6–70.6) | 1,112.1 (931.7–1,279.3) |

Data in parentheses are 95% confidence intervals. Abbreviations: AC=Adenocarcinoma (ICD-10: C15 ICD-O-3: 8140–8141, 8143–8145, 8190–8231, 8260–8265, 8310, 8401, 8480–8490, 8550–8552, 8570–8574, 8576), ASMR= Age-standardised mortality rate per 100,000 population, ASYR= Age-standardised years of life lost rate per 100,000 population, HCC=Hepatocellular carcinoma (ICD-10: C22 ICD-O-3: 8170-8175), SCC=Squamous cell carcinoma (ICD-10: C15 ICD-O-3: 8050-8078, 8083-8084; ICD-10: C21 ICD-O-3: 8050–8076, 8083–8084, 8123-8124).

**Appendix table 3.** Number of preventable cancer deaths and years of life lost in Brazil, China, India, Russia, South Africa, the United Kingdom, and the United States in 2020 due to four major risk factors.

|  | **Brazil** | | **China** | | **India** | | **Russia** | | **South Africa** | | **United Kingdom** | | **Unites States** | |
| --- | --- | --- | --- | --- | --- | --- | --- | --- | --- | --- | --- | --- | --- | --- |
|  | **Deaths** | **YLL** | **Deaths** | **YLL** | **Deaths** | **YLL** | **Deaths** | **YLL** | **Deaths** | **YLL** | **Deaths** | **YLL** | **Deaths** | **YLL** |
| **Alcohol consumption** | 11,900 (7,700–15,900) | 228,800 (150,000–304,300) | 225,700 (129,400–328,300) | 4,094,800 (2,284,800–6,023,100) | 41,600 (17,900–62,300) | 835,800 (358,600–1,254,400) | 19,300 (14,600–23,600) | 317,500 (240,800–386,500) | 2,600 (1,700–3,500) | 46,700 (30,600–62,700) | 6,800 (4,800–8,700) | 98,500 (69,900–125,600) | 18,500 (11,300–25,800) | 318,800 (195,000–442,400) |
| **Tobacco smoking** | 46,600 (36,600–57,100) | 744,600 (573,900–923,200) | 893,900 (752,400–1,031,900) | 13,869,700 (11,429,300–16,225,700) | 110,800 (83,300–140,200) | 1,840,000 (1,342,300–2,373,800) | 79,000 (66,800–90,800) | 1,266,700 (1,064,300–1,460,000) | 8,200 (6,200–10,400) | 134,800 (99,800–174,200) | 45,700 (37,900–53,800) | 607,600 (508,700–706,300) | 159,400 (131,900–187,800) | 2,341,500 (1,956,900–2,729,800) |
| **Excess body weight** | 11,800 (9,200–14,400) | 207,900 (168,200–246,800) | 80,700 (62,300–102,300) | 1,189,500 (939,000–1,470,600) | 8,000 (4,500–12,000) | 158,800 (91,000–234,500) | 32,800 (25,600–39,400) | 441,500 (327,200–546,700) | 3,800 (3,200–4,300) | 57,700 (49,400–65,700) | 10,000 (7,000–12,900) | 120,300 (85,300–152,700) | 60,800 (51,600–69,500) | 930,600 (764,100–1,084,100) |
| **Human papillomavirus** | 10,700 (10,300–11,500) | 253,400 (244,500–267,200) | 62,200 (61,500–63,400) | 1,381,100 (1,368,900–1,400,800) | 89,100 (84,200–96,000) | 1,775,400 (1,683,400–1,907,900) | 9,800 (9,400–10,100) | 210,900 (203,400–216,900) | 6,100 (6,000–6,100) | 141,500 (140,500–143,100) | 2,400 (2,200–2,500) | 47,000 (44,200–49,100) | 10,100 (9,500–10,600) | 206,200 (195,500–213,700) |
| **All four risk factors combined** | 73,500 (59,100–87,800) | 1,291,700 (1,048,400–1,531,100) | 1,144,000 (937,600–1,342,500) | 18,494,500 (14,869,300–21,941,800) | 225,900 (177,100–273,500) | 4,177,000 (3,249,800–5,082,300) | 122,300 (104,000–138,600) | 1,932,000 (1,636,200–2,192,500) | 18,100 (15,500–20,900) | 336,600 (290,800–383,900) | 59,500 (48,700–69,900) | 795,900 (660,000–922,700) | 222,500 (186,800–257,000) | 3,363,100 (2,826,900–3,865,300) |

Data in parentheses are 95% confidence intervals. Abbreviations: AC= Adenocarcinoma (ICD-10: C15 ICD-O-3: 8140–8141, 8143–8145, 8190–8231, 8260–8265, 8310, 8401, 8480–8490, 8550–8552, 8570–8574, 8576), HCC=Hepatocellular carcinoma (ICD-10: C22 ICD-O-3: 8170-8175), SCC=Squamous cell carcinoma (ICD-10: C15 ICD-O-3: 8050-8078, 8083-8084; ICD-10: C21 ICD-O-3: 8050–8076, 8083–8084, 8123-8124), YLL=years of life lost.

**Appendix table 4**. Number and age-standardised rates of years of life lost from preventable cancer deaths in Brazil, China, India, Russia, South Africa, the United Kingdom, and the United States in 2020 due to four major risk factors using the WHO standard life table (sensitivity analysis), by sex.

|  | **Males** | | **Females** | | **Both sexes** | |
| --- | --- | --- | --- | --- | --- | --- |
|  | **YLL** | **ASYR** | **YLL** | **ASYR** | **YLL** | **ASYR** |
| **Alcohol consumption** |  |  |  |  |  |  |
| Brazil | 282,900 (192,700–359,600) | 443.8 (302.8–564.0) | 60,300 (32,200–97,000) | 82.2 (43.8–132.5) | 343,200 (225,000–456,700) | 250.7 (164.7–333.1) |
| China | 5,657,500 (3,173,600–8,210,900) | 974.4 (543.4–1,419.1) | 780,500 (445,400–1,227,800) | 129.5 (73.7–203.9) | 6,438,000 (3,619,000–9,438,700) | 548.2 (305.9–806.8) |
| India | 1,212,800 (527,200–1,769,300) | 333.7 (145.6–485.3) | 178,000 (70,600–313,600) | 50.0 (19.8–88.1) | 1,390,700 (597,700–2,082,900) | 193.5 (83.4–289.0) |
| Russian Federation | 410,700 (322,200–481,600) | 787.5 (617.3–923.8) | 124,700 (83,900–170,000) | 171.5 (114.2–234.8) | 535,400 (406,200–651,700) | 427.8 (324.0–520.9) |
| South Africa | 60,600 (41,200–77,800) | 505.0 (346.5–645.1) | 19,700 (11,500–29,900) | 132.0 (77.2–199.4) | 80,300 (52,700–107,700) | 297.1 (196.6–396.4) |
| United Kingdom | 103,000 (76,200–126,400) | 313.0 (232.4–382.3) | 40,900 (25,900–57,400) | 119.5 (74.5–168.8) | 143,900 (102,100–183,800) | 212.8 (150.7–271.6) |
| United States | 357,200 (220,600–486,200) | 256.3 (159.3–346.6) | 106,700 (63,200–158,000) | 73.5 (42.7–109.7) | 463,900 (283,700–644,200) | 161.5 (99.0–223.6) |
| **Tobacco smoking** |  |  |  |  |  |  |
| Brazil | 756,600 (603,500–908,700) | 1,180.0 (942.3–1,415.7) | 397,900 (290,100–518,600) | 527.2 (383.2–688.3) | 1,154,500 (893,600–1,427,300) | 823.7 (637.3–1,018.1) |
| China | 20,540,700 (17,320,100–23,470,900) | 3,488.5 (2,931.7–3,995.4) | 2,130,900 (1,477,900–2,949,100) | 334.9 (229.7–468.0) | 22,671,600 (18,798,100–26,420,000) | 1,882.9 (1,553.9–2,201.1) |
| India | 2,867,700 (2,191,900–3,516,000) | 819.5 (630.4–1,000.1) | 330,600 (159,100–589,400) | 95.2 (46.0–169.2) | 3,198,300 (2,351,000–4,105,400) | 458.7 (338.8–586.6) |
| Russian Federation | 1,895,900 (1,648,500–2,117,900) | 3,569.4 (3,096.2–3,992.2) | 255,100 (163,100–357,800) | 371.9 (231.7–527.5) | 2,151,100 (1,811,600–2,475,700) | 1,671.7 (1,398.2–1,932.0) |
| South Africa | 168,700 (136,000–199,800) | 1,476.3 (1,201.8–1,736.8) | 66,300 (38,800–102,700) | 449.4 (266.9–689.7) | 235,000 (174,700–302,600) | 897.4 (675.4–1,144.1) |
| United Kingdom | 523,600 (435,300–611,900) | 1,476.3 (1,231.4–1,717.3) | 382,400 (321,900–443,600) | 1,002.9 (841.6–1,163.7) | 906,000 (757,200–1,055,500) | 1,229.0 (1,028.2–1,427.6) |
| United States | 2,080,700 (1,735,300–2,425,500) | 1,367.4 (1,142.0–1,590.7) | 1,413,500 (1,181,100–1,654,200) | 841.1 (697.5–988.1) | 3,494,200 (2,916,400–4,079,700) | 1,090.2 (908.5–1,272.6) |
| **Excess body weight** |  |  |  |  |  |  |
| Brazil | 122,300 (100,700–145,400) | 219.7 (181.1–261.0) | 193,700 (153,300–231,400) | 307.1 (245.9–364.2) | 316,000 (254,000–376,800) | 262.9 (212.5–312.3) |
| China | 941,600 (743,700–1,161,800) | 188.7 (148.7–233.0) | 1,020,100 (797,100–1,277,600) | 191.1 (150.0–238.0) | 1,961,700 (1,540,800–2,439,400) | 191.7 (150.6–238.2) |
| India | 40,500 (19,800–66,300) | 13.0 (6.3–21.4) | 227,100 (132,600–330,100) | 77.2 (45.0–112.2) | 267,600 (152,400–396,500) | 44.6 (25.4–66.2) |
| Russian Federation | 204,900 (164,500–244,000) | 453.0 (363.8–539.2) | 564,500 (409,900–705,300) | 796.3 (554.8–1,015.3) | 769,400 (574,400–949,300) | 660.7 (484.1–823.0) |
| South Africa | 16,900 (14,000–20,100) | 176.9 (145.5–210.0) | 84,500 (72,800–95,600) | 692.8 (598.7–781.7) | 101,500 (86,800–115,700) | 462.3 (396.2–526.1) |
| United Kingdom | 87,900 (57,200–115,300) | 288.7 (206.3–363.1) | 95,500 (72,400–117,900) | 292.0 (212.8–368.3) | 183,400 (129,500–233,200) | 289.6 (210.2–363.8) |
| United States | 425,800 (277,900–555,300) | 363.9 (232.2–479.3) | 954,800 (861,200–1,048,100) | 686.5 (619.3–753.9) | 1,380,500 (1,139,100–1,603,400) | 532.6 (433.1–624.2) |
| **Human papillomavirus** |  |  |  |  |  |  |
| Brazil | 29,000 (17,900–46,600) | 45.1 (27.7–72.7) | 335,100 (332,900–338,100) | 469.3 (466.4–473.2) | 364,100 (350,800–384,800) | 265.7 (256.0–280.8) |
| China | 53,500 (40,300–77,200) | 9.2 (6.9–13.2) | 2,018,900 (2,012,700–2,026,800) | 359.6 (358.6–361.0) | 2,072,400 (2,053,100–2,104,000) | 183.3 (181.7–186.0) |
| India | 246,100 (122,900–427,100) | 68.7 (34.6–118.9) | 2,694,200 (2,662,600–2,735,900) | 753.1 (744.2–764.9) | 2,940,400 (2,785,600–3,163,000) | 406.2 (384.5–437.4) |
| Russian Federation | 46,300 (36,900–53,700) | 90.4 (72.1–105.0) | 289,500 (286,400–292,000) | 502.1 (497.8–505.7) | 335,700 (323,300–345,700) | 308.8 (298.4–317.1) |
| South Africa | 3,100 (2,100–4,800) | 23.9 (15.4–38.0) | 230,900 (230,000–231,800) | 1,428.6 (1,423.6–1,434.6) | 234,000 (232,200–236,600) | 763.5 (757.0–773.0) |
| United Kingdom | 17,600 (15,000–19,500) | 60.2 (51.5–66.6) | 47,000 (45,600–48,200) | 178.1 (173.8–181.5) | 64,600 (60,600–67,600) | 119.8 (113.3–124.7) |
| United States | 67,000 (56,100–74,400) | 49.4 (41.6–54.8) | 224,500 (219,800–228,100) | 182.2 (179.2–184.6) | 291,500 (275,900–302,600) | 116.5 (111.1–120.3) |
| **All four risk factors combined** |  |  |  |  |  |  |
| Brazil | 1,080,100 (852,000–1,290,800) | 1,681.5 (1,327.5–2,008.0) | 895,000 (749,800–1,050,200) | 1,213.2 (1,021.2–1,418.5) | 1,962,600 (1,590,500–2,328,900) | 1,406.9 (1,141.6–1,667.4) |
| China | 24,296,900 (19,803,200–28,238,200) | 4,139.5 (3,358.7–4,825.6) | 5,582,400 (4,515,500–6,883,300) | 925.7 (753.4–1,136.6) | 29,889,800 (24,163,500–35,345,700) | 2,503.3 (2,014.5–2,969.2) |
| India | 4,015,000 (2,742,900–5,120,100) | 1,133.3 (782.0–1,437.0) | 3,267,600 (2,949,000–3,680,100) | 917.2 (826.4–1,034.7) | 7,060,200 (5,499,900–8,580,600) | 991.7 (773.1–1,204.1) |
| Russian Federation | 2,252,800 (1,961,500–2,497,700) | 4,252.7 (3,695.8–4,719.2) | 1,106,900 (873,400–1,327,200) | 1,536.2 (1,213.9–1,840.5) | 3,276,800 (2,777,600–3,716,900) | 2,560.4 (2,164.6–2,907.7) |
| South Africa | 230,100 (182,100–273,700) | 1,987.5 (1,589.0–2,349.2) | 356,900 (323,800–395,200) | 2,293.0 (2,072.6–2,547.3) | 574,900 (495,600–656,400) | 2,065.9 (1,773.0–2,365.3) |
| United Kingdom | 665,000 (543,400–775,900) | 1,892.3 (1,563.9–2,188.7) | 519,400 (436,000–600,600) | 1,414.7 (1,186.0–1,634.5) | 1,184,400 (979,300–1,376,600) | 1,640.7 (1,365.6–1,895.7) |
| United States | 2,648,700 (2,139,600–3,109,100) | 1,781.6 (1,434.8–2,090.2) | 2,367,800 (2,077,500–2,658,400) | 1,468.3 (1,287.4–1,648.8) | 4,993,600 (4,196,800–5,743,400) | 1,606.5 (1,346.8–1,847.9) |

Data in parentheses are 95% confidence intervals. ASYR= Age-standardised years of life lost rate per 100,000 population, YLL = Years of life lost.

**Appendix table 5**. Number and age-standardised rates of years of life lost from preventable cancer deaths occurring between ages 30 and 69 in Brazil, China, India, Russia, South Africa, the United Kingdom, and the United States in 2020 due to four major risk factors using the WHO standard life table (sensitivity analysis), by sex.

|  | **Males** | | **Females** | | **Both sexes** | |
| --- | --- | --- | --- | --- | --- | --- |
|  | **YLL** | **ASYR** | **YLL** | **ASYR** | **YLL** | **ASYR** |
| Brazil | 785,100 (614,800–938,800) | 1,259.4 (987.9–1,504.6) | 708,000 (600,000–822,900) | 1,009.5 (855.0–1,174.2) | 1,481,100 (1,204,300–1,749,700) | 1,113.1 (904.5–1,315.5) |
| China | 18,159,200 (14,511,100–21,329,800) | 3,094.3 (2,458.0–3,648.4) | 3,964,800 (3,265,800–4,829,800) | 692.6 (572.8–841.5) | 22,129,700 (17,638,000–26,365,900) | 1,897.6 (1,505.0–2,268.5) |
| India | 3,414,400 (2,294,200–4,378,200) | 947.9 (643.2–1,208.8) | 2,823,300 (2,548,400–3,177,100) | 793.9 (715.3–895.0) | 6,045,200 (4,679,600–7,363,500) | 843.8 (653.7–1,026.8) |
| Russia | 1,803,900 (1,575,100–1,993,200) | 3,411.0 (2,969.1–3,775.0) | 814,100 (625,300–990,100) | 1,265.2 (984.1–1,529.6) | 2,560,600 (2,162,700–2,906,400) | 2,121.5 (1,786.4–2,412.9) |
| South Africa | 189,400 (149,100–225,500) | 1,555.3 (1,238.4–1,837.5) | 300,900 (274,500–331,800) | 1,935.5 (1,757.5–2,142.4) | 479,200 (414,200–545,700) | 1,693.6 (1,457.5–1,933.5) |
| United Kingdom | 329,300 (277,200–375,700) | 1,274.6 (1,067.9–1,457.9) | 263,000 (220,100–303,600) | 1,011.2 (843.6–1,170.1) | 592,300 (497,300–679,100) | 1,139.6 (953.1–1,310.0) |
| United States of America | 1,681,400 (1,354,100–1,965,400) | 1,311.9 (1,051.5–1,537.5) | 1,441,700 (1,270,400–1,610,500) | 1,113.7 (977.4–1,248.7) | 3,107,800 (2,608,900–3,562,200) | 1,202.1 (1,004.8–1,382.1) |

Data in parentheses are 95% confidence intervals. ASYR= Age-standardised years of life lost rate per 100,000 population, YLL = Years of life lost.

**REFERENCES**

1. Rumgay H, Shield K, Charvat H, et al. Global burden of cancer in 2020 attributable to alcohol consumption: a population-based study. *Lancet Oncol* 2021; **22**(8): 1071-80.

2. Tran KB, Lang JJ, Compton K, et al. The global burden of cancer attributable to risk factors, 2010-19: a systematic analysis for the Global Burden of Disease Study 2019. *The Lancet* 2022; **400**(10352): 563-91.

3. Arnold M, Pandeya N, Byrnes G, et al. Global burden of cancer attributable to high body-mass index in 2012: a population-based study. 2015; (1474-5488 (Electronic)).

4. de Martel C, Georges D, Bray F, Ferlay J, Clifford GM. Global burden of cancer attributable to infections in 2018: a worldwide incidence analysis. *Lancet Glob Health* 2020; **8**(2): e180-e90.

5. Institute for Health Metrics and Evaluation. Global Burden of Disease. 2022. https://www.healthdata.org/gbd/2019 (accessed March 11, 2022).

6. Rumgay H, Ferlay J, de Martel C, et al. Global, regional and national burden of primary liver cancer by subtype. *European Journal of Cancer* 2022; **161**: 108-18.

7. Arnold M, Ferlay J, van Berge Henegouwen MI, Soerjomataram I. Global burden of oesophageal and gastric cancer by histology and subsite in 2018. *Gut* 2020; **69**(9): 1564-71.

8. Bray F, Colombet M, Mery L, et al. Cancer Incidence in Five Continents, Vol. XI. Lyon, France: International Agency for Research on Cancer; 2017.

10. Cao B, Hill C, Bonaldi C, et al. Cancers attributable to tobacco smoking in France in 2015. *Eur J Public Health* 2018; **28**(4): 707-12.
